# Ang2-Targeted Combination Therapy for Cancer Treatment

**DOI:** 10.3389/fimmu.2022.949553

**Published:** 2022-07-08

**Authors:** Na Liu, Mengfang Liu, Shengqiao Fu, Jinglei Wang, Haowen Tang, Adamu Danbala Isah, Deyu Chen, Xu Wang

**Affiliations:** Institute of Oncology, Affiliated Hospital of Jiangsu University, Zhenjiang, China

**Keywords:** Ang2, Targeting therapy, Antiangiogenic therapy, Combination therapy, Cancer development

## Abstract

Angiopoietin-2 (Ang2), a member of the angiopoietin family, is widely involved in the process of vascular physiology, bone physiology, adipose tissue physiology and the occurrence and development of inflammation, cardiac hypertrophy, rheumatoid, tumor and other diseases under pathological conditions. Proliferation and metastasis of cancer largely depend on angiogenesis. Therefore, anti-angiogenesis has become the target of tumor therapy. Due to the Ang2 plays a key role in promoting angiogenesis and stability in vascular physiology, the imbalance of its expression is an important condition for the occurrence and development of cancer. It has been proved that blocking Ang2 can inhibit the growth, invasion and metastasis of cancer cells. In recent years, research has been constantly supplemented. We focus on the mechanisms that regulate the expression of Ang2 mRNA and protein levels in different cancers, contributing to a better understanding of how Ang2 exerts different effects in different cancers and stages, as well as facilitating more specific targeting of relevant molecules in cancer therapy. At the same time, the importance of Ang2 in cancer growth, metastasis, prognosis and combination therapy is pointed out. And finally, we will discuss the current investigations and future challenges of combining Ang2 inhibition with chemotherapy, immunotherapy, and radiotherapy to increase its efficacy in cancer patients. This review provides a theoretical reference for the development of new targets and effective combination therapy strategies for cancer treatment in the future.

## Introduction

**Figure 1:**
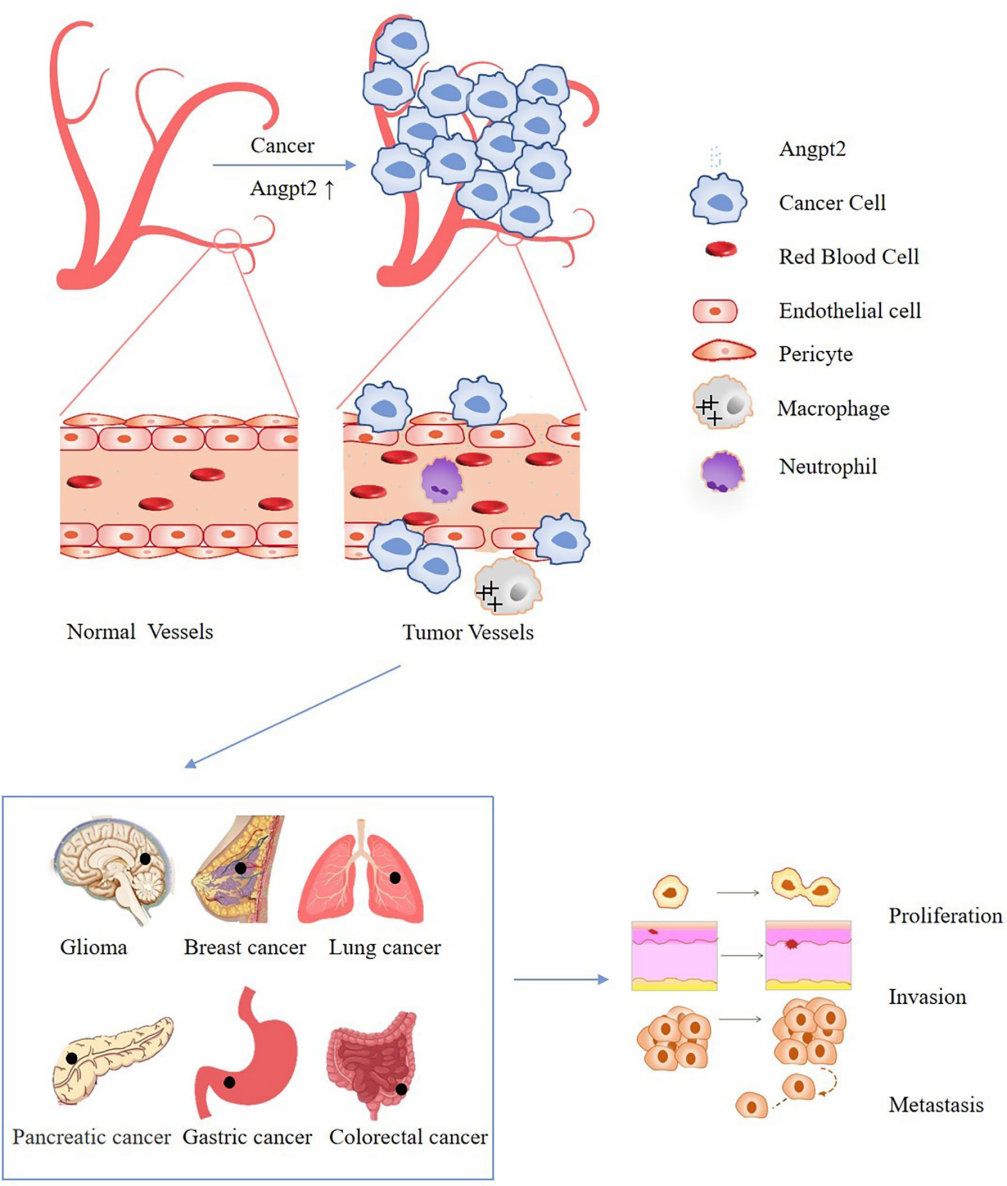
Schematic diagram of the effect of Ang2 dysregulation on cancer cells: The increase of Ang2 in cancer leads to vascular instability, increases leakiness of the vessels, limits immune cell trafficking, and finally promotes the proliferation, invasion and metastasis of cancer.

Tumor cells have the characteristics of infinite proliferation, which will cause most tumor cells to be in hypoxia and nutrient-deficient microenvironment. At this time, the tumor will produce a large number of new blood vessels to provide nutrients and oxygen through the use of blood flow (**Figure 1**) (1–4). It can be seen that the process of new blood vessel formation is necessary for continued tumor growth and progression. antiangiogenic drugs such as bevacizumab and sorafenib are widely used in clinical practice (5, 6). By inhibiting the formation of cancer blood vessels, the blood supply of cancer cells is insufficient, which cannot meet the needs of growth and metastasis, thereby inhibiting the progression of cancer (5, 6). However, the efficacy of these drugs is limited, and their side effects include bleeding, thrombosis, etc. and drug resistance frequently occurs, therefore the discovery of new antiangiogenic targets has become an urgent problem to be solved (7–10). Angiogenesis is coordinated by pro-angiogenic and anti-angiogenic factors, and dysregulation can lead to pathological angiogenesis (8). Ang2 is the ligand of tyrosine-protein kinase receptor Tie-2, which is highly expressed in lung cancer, gastric cancer, colorectal cancer, glioma and other cancers, and also leads to the occurrence and development of cancer by promoting the abnormalization of blood vessels (**Figure 1**) (11–15). Studies have shown that Ang2 is not only a necessary condition for the angiogenesis of cancer cells, but also an indicator of its metastasis, invasion and poor prognosis (12, 15–18). In recent years, Ang2-related inhibitors have been continuously developed (**Table 1**), with the potential for anti-angiogenic and anti-tumor activities (25–29). At present, the combination of anti-angiogenesis therapy with chemotherapy, targeted therapy or immunotherapy has been approved for clinical application and has greatly improved the survival rates of cancer patients (5, 30, 31). Therefore, the prospect of Ang2-targeted combination therapy for cancer treatment is bright. This review mainly discusses the role of Ang2 in various cancers and points out possible potential combination treatment options.

**Table 1:** Targeted inhibition of ang2 in cancer therapy.

Treatment method/drugs	The main function	Cancer type	Stage	References
Nesvacumab (REGN910)	Human anti-ang2monoclonal antibody	Advanced solid tumors	Phase I first in human study	(19)
AMG 780	Angiopoietin 1 and -2 inhibitor	Advanced solid tumors	Phase I first in human study	(20)
AMG 386	Selective angiogenin inhibitors	Advanced solid tumors	Phase I first in human study	(21)
TAvi6	Target VEGF-A and Angiopoietin-2		Preclinical trial	(22)
CVX-241	Target VEGF-A and Angiopoietin-2	Breast cancer	Preclinical trial	(23)
Ang-2-VEGF-A CrossMab (RG7221, vanucizumab)	Target VEGF-A and Angiopoietin-2	Advanced solid tumors	Phase 2	(24)
MEDI3617	A human immunoglobulin G1 (IgG1) kappa monoclonal antibody directed against human angiopoietin-2	Advanced melanoma	Phase I	(25)

## Dysregulated Ang2 and Its Role in Cancers

### Gastric Cancer

The symptoms of gastric cancer in the early stage are not obvious, and most patients are already in the middle and late stages when they first visit the doctor, with a very poor prognosis and high mortality (32–34). Further study on the molecular mechanism of growth, invasion and metastasis of gastric cancer provides a theoretical basis for effective treatment in the future. In recent years, antiangiogenic therapy for gastric cancer has been continuously developed, and many targeted angiogenesis inhibitors are in clinical trials (32, 34, 35).

The occurrence and development of gastric cancer are closely related to the regulation of angiogenesis by microRNA (miRNA) (36, 37). MicroRNAs (miRNAs) are important cancer regulators that function as oncogenes or tumor suppressor genes (37). Studies have shown that miRNAs are involved in the regulation of angiogenesis by regulating the expression of Ang2, and are targets of many cancer treatments (38).

In human umbilical vein endothelial cells and mouse lymph node endothelial cells, miRNA-542-3p inhibits the translation of Ang2 mRNA (37). Researchers added miR-542-3p to a tumor-bearing mice to reduce angiogenesis, tumor growth and metastasis, suggesting that miR-542-3p inhibits tumor progression by weakening the angiogenic activity of Ang2 (37). MiR-218, as a tumor suppressor, inhibits the proliferation and invasion of gastric cancer cells by reducing Ang2 in gastric cancer (39). It is reported that miR-145-5p is low expressed in gastric cancer cells, but Ang2 is highly expressed (40). Further studies have proved that Ang2 is the target of miR-145-5p (40). When miR-145-5p was overexpressed in gastric cancer cells, the expression of Ang2 was significantly down-regulated and inhibiting NOD-LIKE-RECEPTOR signaling pathway which therefore inhibits the proliferation, invasion and metastasis of cancer cells (40). In addition, studies have shown that LINC00184 (the competing endogenous RNA (ceRNA))directly binds to miR-145 and inhibits its expression, to promote the expression of Ang2 and induce the epithelial-mesenchymal transition (EMT) characteristics of gastric cancer cells, and improve the carcinogenesis mediated by Ang2 (41). Dopamine and cAMP-regulated phosphoprotein Mr-32000 (DARPP-32) can induce the expression of Ang2 in gastric cancer cells by regulating signal transducer and activator of transcription 3 (STAT3), promoting angiogenesis and mediating the occurrence and development of gastric cancer (42). Therefore, DARPP-32-STAT3 blocking may prevent the occurrence and development of gastric cancer (42).

From the above discussion, it can be seen that the expression of Ang2 in gastric cancer is regulated not only by miRNA, but also by ceRNA and DARPP-32. In general, all of these have the potential to mediate the proliferation, metastasis and invasion of gastric cancer cells by affecting their blood supply, indicating the feasibility of targeting Ang2 in the treatment of gastric cancer.

### Lung Cancer

Lung cancer, as a disease with a high incidence in the world, has a very complex pathogenesis and mechanism and lacks clear diagnostic indicators and means at the early stage (43–45). Most patients are already at stage III or IV when diagnosed, and the survival rate is very low (46). Therefore, new biomarkers can help to screen lung cancer for early diagnosis and treatment. Ang2 not only participates in tumor angiogenesis but also plays a role in the immune environment of some tumors. Studies have shown that Ang2, Tie2 and Myeloid-derived suppressor cells (MDSC) are involved in the immune escape of non-small cell carcinoma, the collection of clinical data shows that the high expression of ANGPT2/TIE2 + monocytic-MDSC in non-small cell carcinoma is closely related to its poor prognosis (47). Recent meta-data analysis showed that serum Ang2 expression in patients with lung cancer was significantly correlated with the progression and prognosis of lung cancer, and patients with high serum Ang2 expression had a poor prognosis (48). In addition, the abnormal expression of Ang2 is not only related to the stage of lung cancer but also closely related to its invasion, migration and prognosis. After Ang2 interference, the biological characteristics and EMT of lung cancer cells are inhibited, suggesting that Ang2 may be a novel molecular targeted therapy for lung cancer (49, 50). This may solve the current problem of metastatic treatment of lung cancer. After the operation of non-small cell carcinoma, the expression level of Ang2 in the serum of patients was detected to be increased, indicating that the angiogenesis capacity was also increased, which could not only increase the pre-repair of postoperative wound but also promote the distant metastasis and recurrence of cancer (51). This may also be one of the reasons for postoperative recurrence of non-small cell carcinoma. Interestingly, the expression of Ang2 mRNA and protein levels was significantly correlated with the progression and clinical outcome of lung adenocarcinoma. However, this phenomenon was not observed in squamous cell carcinoma (52). It further illustrates the complexity of the regulatory mechanism of Ang2 in cancer.

Lung cancer patients have a high probability of brain metastasis and poor prognosis (53). Exploring the regulatory mechanism of brain metastasis is helpful to identify new therapeutic targets. Studies have shown that the overexpression of a disintegrin and metalloproteinase 9 (ADAM9) can promote the brain metastasis of lung cancer cells, further studies have shown that ADAM9 can promote the vascular remodeling of lung cancer cells and brain metastasis by increasing the expression of vascular endothelial growth factor A (VEGFA), Ang2 and tissue plasminogen activator (PLAT) (54). These findings suggest that targeted inhibition of ADAM9, VEGFA, and Ang2 may be a new effective therapeutic strategy for lung cancer brain metastasis. ADAM9 regulates the expression of angiogenic factor Ang2, thereby controlling vascular remodeling and angiogenesis to regulate lung cancer brain metastasis (54).

VEGFA and ANGPT2 are the targets of anti-angiogenesis therapy, and whether the combined inhibition of ADAM9 with bispecific antibody (A2V CrossMab) against both Ang-2 and VEGF can reduce the morbidity and mortality of lung cancer brain metastasis has not been studied. We look forward to more research on multi-target therapy.

### Glioma

Glioma, as an angiogenesis-dependent tumor, is the most common malignant tumor of the central nervous system. It has strong invasiveness, poor prognosis and easy recurrence after operation (55–57). There is great room for progress in the treatment strategy of glioma. At present, radiotherapy, chemotherapy, surgical treatment and immunotherapy have not solve the key event of high mortality of glioma patients (58). It is necessary to further explore new targets and treatment strategies.

Ang-2 is highly expressed in glioblastoma and is involved in a series of processes such as glioma development, invasion, prognosis and treatment resistance (56, 59–61). Rhythm gene BMAL1 (brain and muscle Arnt like 1) is considered to be a tumor-promoting factor in glioma and plays a key role in the proliferation and migration of glioma cells (59). Studies have shown that BMAL1 is highly expressed in gliomas, and regulates the expression of Ang2 and VEGF by regulating HIF-1a under hypoxia, to participate in the formation of tumor microvessels and peritumoral edema (55). Strangely, when BMAL1 was knocked out, the expression of Ang2 did not change (55). This further indicates the complexity of Ang2 expression regulation. As previous studies have shown, it may be environment-dependent (62). Some studies have shown that the edema of glioblastoma can be alleviated by dexamethasone, and it is suggested that dexamethasone may alleviate brain edema and slow down the growth of gliomas by inhibiting the expression of Ang2 (63). In oligodendroglioma, it was found that under hypoxia, Insulin Gene Enhancer Protein (ISL2) induces angiogenesis by enhancing the expression of Ang2 to promote the growth, malignant transformation and invasion of oligodendroglioma (13, 56). These studies suggest that blocking Ang2-induced angiogenesis through targeted inhibition of ISL2 may be one of the effective strategies for the treatment of oligodendroglioma in the future (13). As an antiangiogenic therapy for glioblastoma, bevacizumab is commonly used, but its drug resistance often occurs. Ang2 is highly expressed in these drug-resistant gliomas (64). It is proposed that the combination of VEGF blocking and Ang-2 inhibition may overcome the resistance of bevacizumab to glioma treatment, suggesting that Ang2 may be a therapeutic target for bevacizumab resistant gliomas (64).

In short, the study on the regulation and expression mechanism of Ang2 in glioma will be beneficial to the future targeted treatment of glioma patients to improve their survival rate and prognosis.

### Colorectal Cancer

Colorectal cancer is considered to be the second most common cancer in the world and the third most common cause of cancer-related death. It has a high risk of recurrence and poor prognosis (65). Although the treatment of colorectal cancer has made progress with the continuous development of immunotherapy and gene-targeted therapy in recent years, the problems of metastatic diseases and drug resistance have not been solved (66). This highlights the necessity to develop new treatment strategies.

Recent studies have reported that RAS-ERK1/2 signaling induces the upregulation of Ang2 and c-x-c motif chemokine receptor 4 (CXCR4) in KRAS-mutated colorectal cancer cells, resulting in liver metastasis (67). The use of ERK inhibitors can downregulate Ang2 and CXCR4 to control the liver metastasis in colon cancer (67). It can be seen that Ang2 plays a key role in liver metastasis of colorectal cancer. Targeted inhibition of Ang2 or RAS-ERK1/2 axis can prevent and treat patients with liver metastasis of colorectal cancer (67). Interestingly, there is also evidence that Ang2 gene deletion may aggravate the progression of liver metastasis in mice (68). On the one hand, Ang2 deletion leads to enhanced bone marrow cell recruitment of granulocyte colony-stimulating factor (G-CSF), which is conducive to more aggressive tumor growth and neoangiogenesis during liver colonization. On the other hand, it is the increase of compensatory VEGF caused by Ang2 deletion, which induces angiogenesis and promotes liver metastases (68). The reasons for the different results of these two studies may be the types of colorectal cancers studied are different, one is KRAS mutated, and the other is common undetected mutations. But both studies suggest that the role of Ang2 depends on the blood vessels of specific organs, as these changes in Ang2 expression were not observed in colorectal cancer lung metastases (67, 68). In addition, according to clinical studies, serum Ang2 levels in patients with colorectal cancer are associated with disease progression. Ang2 was significantly higher in colorectal cancer with peritoneal carcinomatosis than without peritoneal carcinomatosis and was negatively correlated with the survival rate of those patients (16). Additionally, Ang2 is an important predictor of mortality in patients with incurable stage IV colorectal cancer (12). Therefore, it may be a useful prognostic biomarker for colorectal cancer patients (12, 17).

In general, Ang2 is closely related to the occurrence, metastasis, and prognosis of colorectal cancer. Targeting Ang2 or regulating the signaling pathway or factors

of Ang2 in colorectal cancer may effectively inhibit the development of colorectal cancer.

### Breast Cancer

Breast cancer is not only one of the most common malignant tumors in women but also one of the most common causes of death in women (69, 70). At present, there is no effective solution to the distant metastasis, recurrence and treatment resistance of breast cancer.

Clinical studies have shown that Ang2 can not only be used as a diagnostic indicator for the detection of early breast cancer, but also as an evaluation factor for the prognosis of breast cancer (71). In estrogen-deficient conditions, Ang2 promotes survival of estrogen receptor-positive (ER+)breast cancer through integrin 1 (72). More importantly, 2 is highly expressed in the recurrence and metastasis of (ER+) breast cancer patients treated with estrogen antagonists (72). In addition, in estrogen-deficient bone marrow endothelial niche, knockout Ang2 can attenuate tumor cell proliferation. Further research shows that estrogen regulates the proliferation of ER(+) breast cancers by regulating the expression of Ang2 in the bone marrow endothelial niche (72). These experimental results suggest that Ang2 may be a key target for preventing metastatic recurrence of breast cancer in endocrine therapy. At the same time, whether the combined use of estrogen antagonists and Ang2 antagonists can improve the survival rate of patients and reduce metastasis and recurrence requires a lot of research in the future.

### Pancreatic Cancer

Pancreatic cancer is one of the most difficult cancers to treat and the worst prognosis in the world (73). We look forward to more research to explore the pathogenesis of pancreatic cancer and effective treatment options. In recent years, some scholars have proposed that miR-145, as a tumor suppressor of pancreatic cancer, inhibits the angiogenesis, growth and invasion of cancer cells by directly inhibiting the expression of Ang2 (74). It suggests that the inhibition of Ang2 expression by miR-145 indirect targeting may be an effective treatment for pancreatic cancer (74).

Ang2 in both pancreatic cancer and gastric cancer is regulated by miRNA, which mediates the occurrence and development of cancer, further indicating the importance of miRNA in Ang2 regulation.

### Other Cancers

In cerebral cavernous malformation (CCM), CCM3 (also known as PDCD10) gene mutation promotes the progression of CCM (75). The study further showed that CCM3 reduced the secretion of Ang2 and prevented the development of CCM by inhibiting the UNC13B/VAMP3-dependent exocytosis of Ang2 in brain endothelial cells (ECs) (75). On the contrary, when CCM3 is mutated, Ang2 secreted by brain endothelial cells increases, thus accelerating the progress of CCM (75). In conclusion, the Ang2 secretion regulated by CCM3 in endothelial cells may be a new therapeutic target for cavernous malformation.

Kaposi’s sarcoma (KS) is a vascular malignancy associated with Kaposi sarcoma-associated herpesvirus (KSHV) (76). Studies have shown that KSHV contributes to tumor growth by inducing ECs to release Ang2 to promote angiogenesis and inflammatory cell infiltration (77). Ang2 was detected to be highly expressed in KS, and studies further demonstrated that knockdown of Ang2 or use of Ang2 inhibitors AMG-386 and L1-10 blocked angiogenesis and tumor growth in the KS tumor model (77, 78). These findings provide a theoretical basis for the effective combination therapy of KS in the future.

Connective tissue growth factor (CTGF) is a cysteine-rich protein. In osteosarcoma cells, it has been found that overexpression of CTGF can promote the expression of Ang2 and induce angiogenesis of osteosarcoma, providing sufficient blood supply to cancer cells and promoting their metastasis (79).

α-Tocopheryl succinate (TS), an anticancer substance, inhibits tumor angiogenesis by reducing the expression of Ang2 and promoting vascular stabilization in mouse melanoma cells (80). In addition, the detection of Ang2 in melanoma patients showed that the expression of Ang2 in metastatic patients was higher than that in primary tumors (81).

Serum Ang2 is increased in multiple myeloma (MM) patients, especially in advanced patients, and is associated with disease progression (82, 83). It is suggested that Ang2 may be used as a prognostic indicator and a potential therapeutic target for multiple myeloma.

Of course, in addition to the malignant tumors discussed above, whether Ang2 still plays a different role in other tumors needs further exploration and research.

## Targeting Ang2 for Cancer Treatment

The expression of Ang2 is regulated by miRNA, ceRNA and DARPP-32 in gastric cancer, which affects the occurrence and development of gastric cancer. Of course, in addition to the above, whether there will be other factors regulating Ang2 needs further research in the future. In general, targeting miR-542-3p, miR-145-5p, miR-218 LINC00184 and DARPP-32 to inhibit the expression of Ang2 in gastric cancer may be a new strategy for the treatment of gastric cancer, which requires a lot of research in the future. In brain metastasis of lung cancer, Ang2 regulated by ADAM9 plays an important role. Indirect inhibition of Ang2 expression by targeted inhibition of ADAM9 may prevent brain metastasis of lung cancer. In glioma, targeting BMAL1 or ISL2 to regulate the expression of Ang2 may be an effective treatment for glioma in the future. Similarly, the RAS-ERK1/2 signaling pathway in colorectal cancer, estrogen in breast cancer, miR-145 in pancreatic cancer and CCM3, KSHV, CGSF, and TS in other tumors may all be therapeutic targets.

**Figure 2:**
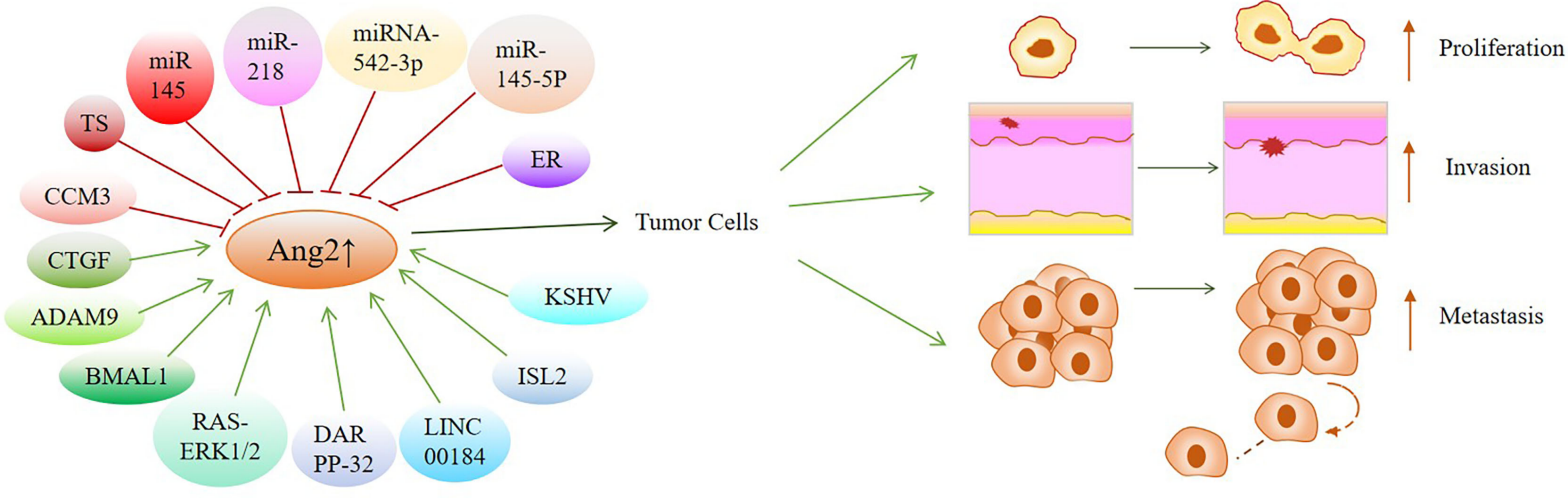
Overview of factors or signal pathways that regulate Ang2 expression in various cancers: These factors or signaling pathways regulate Ang2 expression to mediate the occurrence and development of cancers, which may be potential targets for cancer treatment. Green arrows indicate positive effects. Red perpendicular bars indicate negative effects.

According to the above discussion, there is no doubt that the expression of Ang2 in cancer is regulated by various factors (**Table 2**). We fully introduced the regulatory mechanism of Ang2 expression in different cancers and proposed potential targets to inhibit the occurrence and development of cancers by controlling Ang2 expression (**Figure 2**).

**Table 2:** Dysregulated Ang2 in cancer.

Cancer type	Regulatory factors	Effects on the expression of Ang2	Cancer Development	References
Gastric cancer	miRNA-542-3p	↓	Inhibit cancer cell proliferation, migration	(37)
	miR-218	↓	Inhibit cancer cell proliferation and invasion	(39)
	miR-145-5p	↓	Inhibit cancer cell proliferation, migration and invasion	(40)
	LINC00184	↑	Induced EMT characteristics of gastric cancer cells	(41)
	DARPP-32	↑	Promotes cancer cell proliferation, invasion and migration	(42)
Lung cancer	ADAM9	↑	Promotes cancer cell migration	(54)
Glioma	BMAL1	↑	Promotes cancer cell proliferation, invasion and migration	(55)
	ISL2	↑	Promotes cancer cell proliferation, invasion and migration	(13)
Colorectal cancer	RAS-ERK1/2	↑	Promotes cancer cell migration	(67)
Breast cancer	Estrogen	↓	Inhibit cancer cell proliferation	(72)
Pancreatic cancer	miR-145	↓	Inhibit cancer cell proliferation and invasion	(74)
Cerebral cavernous malformation (CCM)	CCM3	↓	Inhibit cancer cell proliferation, migration and invasion	(75)
Kaposi’s sarcoma	Kaposi sarcoma-associated herpesvirus (KSHV)	↑	Promotes cancer cell proliferation, invasion and migration	(77)
Osteosarcoma	Connective tissue growth factor (CTGF)	↑	Promotes cancer cell migration	(79)
Melanoma	TS	↓	Inhibit tumor angiogenesis	(80)

## Progress of Targeted Inhibition Ang2 Combined With Other Therapies in Cancer Treatment

### Chemotherapy

Chemotherapy combined with anti-angiogenic therapy is constantly being developed and has been applied to colon cancer, gastric cancer and other cancers (5, 34). It is reported that the treatment effect of patients can be improved by adding bevacizumab to the chemotherapy regimen of metastatic colorectal cancer (such as 5-FU and irinotecan) to inhibit vascular endothelial growth factor (VEGF) (84).

However, after receiving bevacizumab anti-angiogenesis treatment, patients will develop drug resistance, and the increased expression of Ang2 is closely related to patients’ anti-bevacizumab resistance (84). Further studies have shown that high Ang-2 levels are associated with adverse clinical outcomes in patients with colorectal cancer treated with bevacizumab. Inhibition of Ang2 will increase resistance to VEGF signal-targeted therapy (84). Therefore, a bispecific antibody (CrossMab), that is, the combined inhibition of VEGF and Ang2, was proposed, which is currently considered to improve anti-angiogenic therapy (22). In addition, a bispecific antibody targeting VEGF and Ang-2 (CrossMab) in combination with chemotherapy was also evaluated in a model of drug-resistant colorectal cancer. The results showed that the bispecific antibody (CrossMab) combined with chemotherapy including 5-FU and irinotecan exhibited better therapeutic effect and addressed the limitations of single antiangiogenic therapy and chemotherapy (84). More surprisingly, studies have shown that bispecific antibody (A2V) combined with Ang2 and VEGFA blockade is more effective than monotherapy in renal cell carcinoma, metastatic breast cancer, and pancreatic neuroendocrine tumors (84–86).

MEDI3617, a selective Ang2 inhibitor, neutralizes Ang2 by blocking the interaction between Ang2 and Tie2 receptors and inhibits angiogenesis and tumor growth (26). Treating mice with MEDI3617 can inhibit angiogenesis in mouse tumor models. The combination of MEDI3617 with chemotherapy or bevacizumab leads to delayed tumor growth (26). In human tumor xenograft models, the application of Ang2 inhibitor combined with paclitaxel or carboplatin in advanced solid tumors is currently in phase I clinical trial (27). Overall, these clinical findings suggest that Ang2 as an anti-angiogenesis therapeutic target, combined with chemotherapy plays a synergistic role in the treatment of cancer.

### Immunotherapy

Ang2 reduces the ability of the immune system (mainly T cells) to recognize and attack tumors by acting on immune cells (9, 87, 88). In addition, Ang2 can prevent immune cells from infiltrating into the tumor by destroying the stability of tumor blood vessels, leading to vascular abnormalities and destroying blood flow (**Figure 1**) (9, 87, 88). Since Ang2 has immunomodulatory effect, its importance in immunotherapy can’t be ignored. Immunotherapy, as a treatment for cancer, has emerged in recent years, but it will also be ineffective in some tumors. Research is also constantly adding how to better improve immunotherapy. Studies have shown that anti-angiogenesis therapy can improve the effect of immunotherapy (7, 89–93). Targeted inhibition of VEGF and Ang2 reduces angiogenesis and normalizes abnormal vasculature (92). More importantly, it can improve tumor immune response and patient prognosis (92). In the mouse model of glioblastoma, the combination of dual anti-angiogenesis and PD-1 checkpoint therapy significantly prolonged the survival time and normalized the vascular system of glioblastoma mice compared with anti-angiogenesis therapy alone (61). At the same time, the increase of T cells not only reduced immunosuppression but also induced stronger antitumor immune response and reduced glioma edema (61). Combined therapy not only eliminates the side effects of single antiangiogenic therapy but also enhances antitumor immunity through the synergistic effect of VEGF/Ang2 and PD-1 blockers (61, 86, 94). In addition, recent clinical trials have proved that MEDI3617 combined with tremelimumab (an IgG2 monoclonal antibody blocking cytotoxic T-lymphocyte-associated protein - (CTLA-4)) is safe in the treatment of patients with advanced melanoma, which significantly reducing the toxicity and side effects of single treatment (25). These studies provide strong support for co-targeting of angiogenesis and immune checkpoints in cancer therapy.

### Radiotherapy

The effects of ionizing radiation on angiogenesis are complex, mainly depending on the dose of radiation (95). After locally advanced rectal cancer patients underwent low-dose ionizing radiation therapy at a dose below 0.8 Gy (called LDIR), the endothelial cell ECs around the tumor were activated, thereby up-regulating the expression of several pro-angiogenic genes such as Ang2, VEGFR1, VEGFR2, Induction of peritumoral angiogenesis (96, 97). In the early days, some scholars proposed the combined use of radiation and anti-angiogenic agents to treat cancer (98, 99). Studies have confirmed that the combination of antiangiogenic therapy and radiotherapy in head and neck squamous cell carcinomas (HNSCC) and nasopharyngeal carcinoma can not only overcome the side effects of separate treatment but also improve the curative effect (14, 100, 101). Interestingly, recent studies have shown that overexpression of Ang2 in mouse glioma models combined with radiochemotherapy can prevent the recurrence of glioblastoma (60). Therefore, the role of Ang2 in cancer treatment needs to be further explored to play a more accurate targeted therapy.

In general, targeting Ang2 inhibits the formation of blood vessels in cancer cells, resulting in insufficient blood supply to cancer cells, unable to meet the needs of growth and metastasis, thereby inhibiting cancer progression, while also increasing the sensitivity of radiotherapy and improving efficacy (14). However, there are many problems in the combined use of anti-angiogenic therapy and radiotherapy, such as the order of use of the two, the dose and time of radiation, the amount of vascular inhibitor, the route of administration, etc. These problems require a lot of clinical trials to study.

## Conclusion

Antiangiogenic therapy is one of the important means of tumor treatment at present (102) It does not only block the nutrition and oxygen required by tumor cells but also normalize abnormal blood vessels and increase the sensitivity of radiotherapy and chemotherapy (9, 92). Bevacizumab, erlotinib, apatinib and other drugs are widely used in clinic, especially in combination with chemotherapy drugs, which significantly improves the curative effect in the treatment of colorectal cancer, small cell lung cancer and other cancers, but its side effects and drug resistance have not been solved (103–105). In recent years, Ang2 has become a new target of anti-angiogenesis therapy. In addition, Ang2 inhibition combined with chemotherapy, radiotherapy and immunotherapy has been proved to improve the effect of tumor treatment and overcome the limitations of single treatment. But there are still a lot of problems to be solved. For example, the sequence, duration, route of administration and dosage of the combination therapy. The expression of Ang2 is regulated by different mechanisms in different tumors, and even in different types of the same tumor. A better understanding of the mechanism of high Ang2 expression in cancer and the vascular changes mediated by it will help to address problems with current anti-angiogenesis in cancer therapy. Here, we mainly introduced that the expression of Ang2 in lung cancer, gastric cancer, glioma, colorectal cancer, breast cancer and other cancers is regulated by relevant signal pathways or factors, and proposed the possibility of targeting the inhibition of Ang2 with the signal pathways or factors that regulate the expression of Ang2. Therefore, in the future, we should further explore the role of Ang2 in cancer to maximize the efficacy for cancer patients. In addition, the optimal combination of targeting the inhibition of Ang2 with chemotherapy, radiotherapy, and immunotherapy would require a concerted effort.

Ang2 is not only involved in the pathology of many diseases, but also related to anti- angiogenesis and drug resistance, making it an ideal target. However, the mechanism of Ang2 in different cancers, even in different stages of the same cancer, needs further study.

## Author Contributions

DC, XW and NL provided direction and guidance throughout the preparation of this manuscript. NL, ML and SF wrote, edited and revised the manuscript. HT, JW and AI participated in the collation of article tables. All authors have read and approved the final manuscript. All authors contributed to the article and approved the submitted version.

## Funding

This study was supported by the National Natural Science Foundation of China No: 32170910; Natural Science Foundation of Jiangsu Province: BK20211124; Zhenjiang Key Research and Development Program: SH2021037.

## Conflict of Interest

The authors declare that the research was conducted in the absence of any commercial or financial relationships that could be construed as a potential conflict of interest.

## Publisher’s Note

All claims expressed in this article are solely those of the authors and do not necessarily represent those of their affiliated organizations, or those of the publisher, the editors and the reviewers. Any product that may be evaluated in this article, or claim that may be made by its manufacturer, is not guaranteed or endorsed by the publisher.

## Abbreviations

Ang2, Angiopoietin-2; miRNA, microRNA; miRs, MicroRNAs; ceRNA, Competing endogenous RNA; EMT, Epithelial-mesenchymal transition; DARPP-32, Dopamine and cAMP-regulated phosphoprotein Mr 32000; STAT3, Signal transducer and activator of transcription 3; MDSC, Myeloid-derived suppressor cells; ADAM9, A disintegrin and metalloproteinase 9; VEGFA, Vascular endothelial growth factor A; PLAT, Plasminogen activator; BMAL1, Brain and muscle Arnt like 1; ISL2, Insulin Gene Enhancer Protein; CXCR4, C-x-c motif chemokine receptor 4; G-CSF, Granulocyte colony-stimulating factor; ER, Estrogen; ER+, Estrogen receptor-positive; CCM, Cerebral cavernous malformation; Ecs, Endothelial cells; KS, Kaposi’s sarcoma; KSHV, Kaposi sarcoma-associated herpesvirus; CTGF, Connective tissue growth factor; TS, α-Tocopheryl succinate; MM, Multiple myeloma; VEGF, Vascular endothelial growth factor; LDIR, Low-dose ionizing radiation; HNSCC, Head and neck squamous cell carcinomas; CTLA-4, cytotoxic T-lymphocyte-associated protein -4.
